# Associations between sleep deficit and academic achievement - triangulation across time and subject domains among students and teachers in TIMSS in Norway

**DOI:** 10.1186/s12889-022-14161-1

**Published:** 2022-09-21

**Authors:** Frøydis N. Vik, Trude Nilsen, Nina C. Øverby

**Affiliations:** 1 Center for Lifecourse Nutrition, Department of Nutrition and Public health, University of Agder, Post box 422, 4604 Kristiansand, Norway; 2 Department of Teacher Education and School Research, University of Oslo, Niels Henrik Abels hus, Moltke Moes vei 35, 0851 Oslo, Norway

**Keywords:** Adolescents, Cognitive outcomes, Norway, School achievements, Sleep, Sleep deficits, Sleepiness, TIMSS

## Abstract

**Background:**

Sufficient sleep is important to an individual’s health and well-being, but also for school achievement among adolescents. This study investigates the associations between sleepiness, sleep deficits, and school achievements among adolescents.

**Methods:**

This trend study involved a representative sample of Norwegian adolescents based on the “Trends in International Mathematics and Science Study” (TIMSS), *N* = 4499 (2015) and *N* = 4685 (2019) and their teachers. The students were 9th graders from a Norwegian compulsory secondary school. The survey included questions on students’ *sleepiness* as students reported in 2019 and *sleep deficits* among students that limited teaching in class as their teachers reported in 2015 and 2019. Regression, triangulation, and mediation analyses were used. Mplus was used to perform the statistical analyses.

**Results:**

The results revealed significant negative associations between sleep deficits and school achievements, adjusted for gender, socioeconomic status (SES), and minority status among Norwegian 9th graders. These results were found for both mathematics and science achievements in 2015 and 2019. Sleepiness that the students reported was negatively associated with school achievements in 2019. Trend and mediation analyses showed that sleep deficits explained 18 and 11% of the decrease in mathematics and science achievements, respectively, from 2015 to 2019.

**Conclusions:**

Sleep deficits were associated with school achievements in mathematics and science among Norwegian 9th graders. Mediation analyses revealed that sleep deficits explained a significant part of the decline in academic achievements. Insufficient sleep may have negative public health implications and influence adolescents’ academic achievements and competences, and should therefore be discussed in both the educational and health systems.

## Background

Sufficient sleep is fundamental to a person’s health and well-being [1]. Sleep provides optimal circumstances for cognitive development and is thought to play a crucial role in memory consolidation, which is essential for academic achievement [2]. If academic success deteriorates because students feel sleepy, the consequences could be dire with regards to competition for further studies and future jobs. Moreover, lack of sleep could lead to inequality among students, as students who feel tired may lag behind other students in academic achievements [3].

While there are studies that address the association between sleep and academic achievements internationally, there is a need for new knowledge due to lack of research assessing this relationship in the Nordic setting [4]. This is especially true because there seems to be a change in young people’s sleep patterns [5] and a negative trend in academic achievement in mathematics and science reported for Norwegian students [6]. A trend analysis of sleep as it relates to student achievement is needed. Further, there are important methodological challenges in the current literature, such as a lack of representative samples [7]. Having a representative sample gives outcomes that are more likely to resemble the population in general and yields more trustworthy and relevant results. Inferences made from representative samples are, furthermore, generalizable and useful for educational policy.

### The Norwegian school system and factors influencing students’ academic achievements

In Norway, 96% of all students attend public schools [8]. The children start school when they are 6 years old. All Norwegian children and adolescents attend mandatory school for 10 years with a final examination that is equal for everyone. After that, most students choose academic tracks, preparing for university studies (3 years) or vocational training (4 years).

Currently, there is extensive research investigating the factors that promote students’ school outcomes [9]. These are typically school factors, such as school climate [10, 11], teacher competence [12, 13], teacher self-efficacy and beliefs [14], and the teacher’s instruction [15–17]. Studies on student behavior and disposition, such as diet or sleep [18], rarely relate these to academic achievement in Nordic countries. A recent study, however, revealed a positive association between breakfast intake and academic achievements among Norwegian adolescents [19]. Educational policy needs to know of all the factors that promote positive student learning outcomes. Hence, there is a need to investigate the relationship between student behavior and dispositions and student academic learning outcomes; among these, a fundamental factor to learning is sleep [20].

### Trends in sleep duration among students over the last decade

A systematic review reported the sleep duration of children aged 5–18 years over a period of 103 years (1905 to 2008) [21]. Data were available on 690,747 children/adolescents from 20 countries and the results indicated a decrease of more than 1 hour of sleep per night over the study period. The greatest rate of decline in sleep occurred for older children, especially boys, and on schooldays, with the results varying according to region [21]. We know from a more recent study [22] that sleep duration among adolescents in Norway has been reduced to 6.25 hours on school nights, while the recommendation for the 14–17 years age group is 8–10 hours of sleep [5]. The adolescents may then catch up on their sleep during the weekends, indicating a sleep deficit of about 2 h [22]. The authors reported that it was common to use electronic devices in bed after bedtime, and increased use correlated with a shorter duration of sleep and increased sleepiness during the school day. This is supported in a review of 36 studies with school-aged children and adolescents that showed that electronic media use was significantly related to delayed bedtime and shorter total sleep time [23]. Mechanism for this might be that media use reduces sleep, increases arousal, and bright light exposure from screens might delay melatonin secretion, and thereby delay sleep rhythms [24].

### Sleep and school achievements

Positive associations between appropriate amounts of sleep and academic achievements have been demonstrated [25]. Insufficient sleep among adolescents has also been associated with weakened emotional-behavioral regulation and poor academic achievement [24]. A meta-analytic review of one longitudinal and 16 cross-sectional studies found that short sleep duration, poor sleep quality, and sleepiness were all negatively related to school achievements in children and adolescents [26]. The effect was strongest for sleepiness, followed by sleep quality and sleep duration [26]. Adelantado-Renau et al. found that self-reported sleep quality among adolescents was positively associated with academic performance in Spanish students [27]. Boschloo et al. investigated the relationship between sleep and school achievements among 11–18-year-olds and they found that sleepiness measured by “I feel sleepy during the first hours at school” predicted both school grades and self-reported school achievements [28]. Further, they suggested that sleepiness may be a better predictor of objective school achievements than both sleep quality or sleep duration, which were also used as measures, because reduced sleep quality may give rise to sleepiness in the first hours of school which results in lower school achievements [28]. Another main problem related to sleepiness is the tendency to fall asleep and nap during school hours, with an obvious negative impact on academic achievements. There are also other important pathways to consider; for example, sleep deficits are also associated with poorer executive functioning [29, 30], which could affect concentration in school and the practice of effective study habits.

However, while insufficient sleep seems to have a negative impact on cognitive outcomes, different effects have been demonstrated for different groups of adolescents. The negative effects of insufficient sleep on emotional-behavioral regulation and academic achievement are more pronounced in adolescents from families with lower socioeconomic status (SES) [24]. Lower SES has had an association with lower total sleep duration, when objectively measured [31]. The negative effects that inadequate sleep has on academic achievement was also reported as comparatively greater in children and adolescents from lower SES families [32]. Studies examining sleep deficits among adolescents and cognitive outcomes, should hence control for SES.

### Aim

To address the current research gaps, we utilize the most recent data available from the Trends In Mathematics and Science Study (TIMSS) of Norwegian 9th graders and their teachers in 2015 and 2019 [6, 33], and ask the following research questions:

1. How has teachers’ perceptions of students’ sleep deficit changed between 2015 and 2019?
2. a. What are the associations between students’ reports on sleepiness at school and their academic achievements in mathematics and science in 2019?
  b. What are the associations between teachers’ perceptions of students’ sleep deficits and the students’ academic achievements in mathematics and science in 2015 and in 2019?
3. To what extent may students’ sleep deficits explain changes in their achievements in science and mathematics from 2015 to 2019?

The reason we include both mathematics and science outcomes, the students’ and teachers’ reports, and two time points is to triangulate and thus validate our findings in order to provide more robust inferences. We hypothesize that students’ sleepiness at school is negatively associated with academic achievements in mathematics and science.

## Methods

### Design and setting of the study

The current study is based on data from the international, large-scale assessment study, Trends in International Mathematics and Science Study (TIMSS); TIMSS 2019 and TIMSS 2015. The TIMSS follows the principles of the Declaration of Helsinki. The study is repeated every fourth year, and Norway has participated since 1995.

The TIMSS is a trend study, meaning that the scores on mathematics and science are comparable across time (for more on this, see Martin, von Davier [34]). The mean achievement score is set to 500 with a standard deviation of 100, according to the cycle of 1995 [34].

### Participants

The present study includes representative samples at the national level of Norwegian 9th graders who participated in TIMSS 2015 and 2019, as well as their mathematics and science teachers. The TIMSS implements a two-stage random sample design, with a sample of schools drawn as a first stage and two intact classes of students selected from each of the sampled schools as a second stage (if the school only has one class, then one class is sampled) [33].

Table 1 provides descriptive statistics on the samples and shows how Norwegian students’ achievements in mathematics and science decreased from 2015 to 2019 by 9 points and 13 points, respectively.

**Table 1:** Descriptive statistics (standard error in parentheses)

	2015	2019
Number of students	4499	4685
Number of schools	150	154
Mathematics achievement	512 (2.3)	503 (2.4)
Science achievement	509 (2.8)	495 (3.1)
Percentage of girls	50.0%	49.2%

### Measures

The TIMSS measures mathematics and science competence in grades 4 and 8 (for Norway: grades 5 and 9), and includes contextual information from student, parent, teacher, and principal questionnaires [33]. In science and mathematics, there are more than 200 items covering all content dimensions as well as all cognitive dimensions (knowing, applying, and reasoning) [33]. About half of the items are multiple choice and the rest are open response items. The trend items constitute about half of the items, are not publicly available, and are the same from one cycle to the next.

The present study also includes contextual information from student and teacher questionnaires.

#### Sleepiness

In the TIMSS 2019 student questionnaire, the students were asked, “How often do you feel this way when you arrive at school?” The students then rated how often they felt sleepy. The response options were “Never”, “Sometimes”, “Almost every day”, and “Every day”. In the TIMSS 2015 student questionnaire, the students were not asked about sleepiness.

#### Sleep deficit

The questions in the teacher questionnaires of 2019 have not changed since 2015. The teachers were asked, “In your view, to what extent do the following limit how you teach this class?” Both the mathematics and science teachers rated the statement, “Students suffering from not enough sleep”. The response options were, “Not at all”, “Some”, and “A lot”. In other words, this is an indirect measure of students’ sleep deficit.

The two measures of sleepiness and sleep deficit have been used in several cycles of TIMSS across more than 50 countries. It is further piloted before every cycle.

#### SES, gender, and minority status

Socioeconomic status, gender, and minority status (non-native Norwegian speakers) were used as control variables since there is literature to support differences in these variables in sleep-related issues, e.g., sleep deficits have been shown to be more prevalent among minorities and socially disadvantaged groups [35–38]. Socioeconomic status was measured as a composite variable made by TIMSS and using item response theory (see https://timss2019.org/reports/home-educational-resources-8/ and TIMSS technical report: https://timssandpirls.bc.edu/timss2019/methods/index.html?_gl=1*1vdknef*_ga*MjEzNTQyNzE3Mi4xNTM0NjY2Mzcx*_ga_L2FMXN42HR*MTY1MzU1NjA0NS4zMC4wLjE2NTM1NTYwNDUuMA). Socioeconomic status is rated by students and consists of parents’ education, how many books there are in the home, and the number of home study supports (such as having their own room). Minority status is measured by students’ ratings of how often they speak Norwegian at home, with the following response options: “Never”, “Sometimes”, “Almost always”, and “Always”, coded from 0 (“Never”) to 3 (“Always”). The gender variable is coded 0 for girls and 1 for boys.

To summarize, we have two indicators for sleep deficits: 1) reported by students in 2019, and 2) by teachers in 2015 and 2019.

### Statistical analyses

The data from 2015 and 2019 was merged into one SPSS file. Mplus version 8 [39] was used to conduct a two-step regression analysis. Robust maximum likelihood was used to account for missing data. To take into account the hierarchical design of the data, where students are nested within classes and classes within schools, we used the Mplus option “type = complex”, where the clustering variable is IDCLASS (the unique class identification). The class weights and plausible values for mathematics and science were included according to recommended procedures [40].

In Step 1, regression models were used to estimate the relationship between the predictors (sleep deficits and sleepiness) and student outcomes. This model is denoted as Model 0. We then control for SES, gender, and minority status, and this model is denoted as “Full model”. We triangulated the results in three ways, investigating the relationship between predictors and student outcomes by: 1) using both student-reported data and teacher-reported data, 2) using student outcome in science and mathematics, and 3) using data from 2015 and 2019 (the latter was only done for sleep deficit, as sleepiness was only measured in 2019). This way, we aimed to ensure the reliability and validity of the results by using data based on different respondents, different outcomes, and at two different time points.

**Fig. 1:**
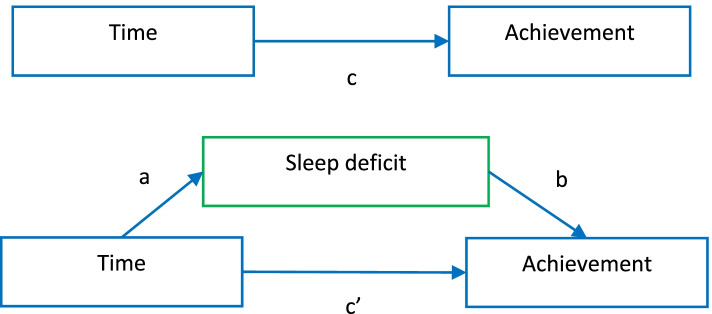
Mediation model

In Step 2, we used trend mediation analyses that resemble those of quasi-longitudinal models, only with trend data [41, 42]. We investigated whether sleep deficits may explain changes in achievements in mathematics and science from 2015 to 2019. Sleepiness was not included in these analyses as this was only measured in 2019. We did this by investigating whether the predictors mediate the effect of time on achievement (see Fig. 1).

A dummy variable for time—coded 0 for the 2015 cycle and 1 for 2019—was created. The effect of time on achievement was expected to be negative for both science and mathematics since we already knew that these achievements had decreased [33]. The unstandardized regression coefficient for the effect of time on achievement (c) was expected to be around − 13 for science, and − 9 for mathematics as reported in the TIMSS international report [32]. The effect of time on sleep deficits, as reported by teachers (path a), will be negative if the problem is bigger in 2019 than in 2015. The effect of sleep deficits on achievement will be positive if *less* sleep deficits have a positive effect on achievement (path b). The direct effect of time on achievement (path c`) is expected to decrease if sleep deficit mediates the time changes in achievement. The indirect effect of sleep deficit (a times b, not shown in Fig. 1) reflects how many points of the decrease in achievement are explained, or mediated, by sleep deficit. In this model, the indirect effect is the most interesting as it provides answers to research question 3. Socioeconomic status, gender and minority status were not included in this model, as they did not change from 2015 to 2019 [33, 41].

## Results

### Research question 1: how have teachers’ perceptions of students’ sleep deficits changed between 2015 and 2019?

Since students only reported this in 2019, we examined the teachers’ reports of changes in the way the students’ sleep deficits limit their teaching from 2015 to 2019.

With regards to sleep deficits, 37.8% of science teachers reported that students’ sleep deficits did not limit their teaching (“not at all”) in 2015, while only 23.6% said the same in 2019. This is a decrease of about 14% between 2019 and 2015, meaning that this problem has increased. Similarly, there were 10% less mathematics teachers who reported that sleep deficit was an issue (“not at all”) in 2015 as compared to 2019. Hence, the percentages of mathematics and science teachers who report that this is an issue (“Some” and “A lot”) has increased from 2015 to 2019.

### Research question 2: the relationship between sleepiness and sleep deficits on academic achievement

The relationship between sleepiness (reported by students) and outcomes in 2019 are shown in Table 2. Before controlling for SES, gender, and minority status, the effects of sleepiness on mathematics and science achievements are significant. Being less sleepy is associated with an increase of about 8 score points in mathematics achievement (unstandardized regression coefficient). One year of schooling in lower secondary school results in about 20 score points in Norway [6, 43]. Hence, 8 score points are a little less than half a year of schooling. When controlling for SES, gender, and minority status, the regression coefficient remains at about 8. For achievements in science, the regression coefficient was about 5 after taking the control variables into account. Hence, the results show that feeling sleepy at school is associated with lower achievements in mathematics and science.

**Table 2:** Relationship between sleepiness and student achievement in 2019 based on students’ reports

2019	Sleepiness	SES	Gender	Minority Status
Model 0 Math	8.20* (0.09)			
Full model Math	8.06* (0.09)	48.96** (0.31)	2.19 (0.01)	5.89* (0.07)
Full model Science	5.03* (0.05)	60.79 **(0.33)	8.35* (0.05)	12.62** (0.10)

* denotes *p* < .05, ** denotes *p* < 0.001. Standardized regression coefficients are in parentheses

The relationship between teachers’ reports on sleep deficits and outcomes in 2015 and 2019 are presented in Table 3. Before controlling for SES, gender, and minority status, the effect of the predictor (“Sleep deficits” reported by teachers) on mathematics achievement is significant in both 2015 and 2019. An increase in sleep deficits is associated with a decrease of about 10 score points in mathematics in 2015 and 9 in 2019. Sleep deficits have a smaller regression coefficient (after controlling for SES, gender, and minority status), but still account for approximately 8 points in 2015 and 7 points in 2019. Similar results are found for achievement in science, albeit with slightly higher regression coefficients for the predictor in 2015 and lower in 2019. In general, the results indicate that sleep deficit was associated with a decrease in student achievement in mathematics and science in both cycles; 2015 and 2019.

**Table 3:** Relationship between sleep deficits and student achievement in 2015 and 2019 based on teachers’ reports

	Sleep deficit		SES		Gender		Minority Status	
	2015	2019	2015	2019	2015	2019	2015	2019
Model 0 Math	9.94* (0.08)	8.46* (0.07)						
Full model Math	7.79* (0.06)	6.77* (0.05)	43.87** (0.31)	50.99** (0.32)	4.60 (0.03)	5.34 (0.03)	11.55* (0.10)	4.61* (0.04)
Full model Science	9.86* (0.07)	5.96* (0.04)	52.74** (0.33)	61.50** (0.35)	9.91* (0.06)	10.64* (0.06)	19.51** (0.15)	12.01* (0.10)

* denotes *p* < .05, ** denotes *p* < 0.001. Standardized regression coefficients are in parentheses

### Research question 3. Trend

In research question 3, we examined the extent to which sleep deficits may explain changes in achievements in science and mathematics from 2015 to 2019. The trend analyses showed that the effect of time on science and mathematics achievement was 13.01 and 8.42 score points (unstandardized), respectively. This was before adding the predictor. This means that students’ science achievements decreased by 13 points, and mathematics decreased by about 8 points. This is in line with the estimates of TIMSS [33]. The results from the mediation models for science and mathematics achievement are presented in Table 4. In these models, we examined whether sleep deficits may mediate the effect of time on achievement. This could be rephrased into whether sleep deficits could explain the changes in adolescents’ school achievements. Sleep deficits explained about one-and-a-half points (the indirect effect) in both subjects, which is about 11% of the total decline in science achievement and 18% in mathematics achievement.

**Table 4:** Predictor (sleep deficit) mediating the effect of time changes on student achievements

Subject domain	Effect of time on sleep deficits	Effect of sleep deficits on achievement	Effect of time on achievement	Indirect effect
In science	−0.14* (− 0.12)	10.15** (0.07)	− 11.78** (− 0.07)	−1.40* (− 0.01)
In mathematics	−0.15* (− 0.13)	10.00** (0.08)	− 5.32* (− 0.04)	− 1.48* (− 0.01)

* denotes *p* < .05, ** denotes *p* < 0.001. Standardized regression coefficients are in parentheses

## Discussion

In this study, we used data from TIMSS Norway and found that the teachers’ perceptions of students’ sleep deficits worsened from 2015 to 2019. More specifically, the teachers reported on how sleep deficits among students limited the ability to teach them in class, and the results indicate that it has become more challenging. This predictor had a significant association to school achievements in both mathematics and science among Norwegian 9th graders, both in 2015 and 2019. Students’ ratings supported this finding: students reported a positive association between feeling less sleepy and their achievements in mathematics and science in 2019. Further, mediation analyses showed that sleep deficit explains the decrease in achievement (1.4 points in science and 1.5 in mathematics).

The results were triangulated across subject domains, students’ and teachers’ ratings, and time. Alignments in terms of changes from 2015 to 2019 were found in variables related to sleepiness and sleep deficits as well as their effects on school achievement. The associations were still significant when gender, SES, and minority status were adjusted for in the analyses. Our results were also triangulated using different methodology (descriptive statistics, analyses for separate samples, and trend study approaches on merged samples). These methods are robust and complex, and in addition, the study sample is representative. Hence, the inferences drawn are valid and reliable.

Our results showed that the teachers reported enhanced sleep deficits at school in 2019 as compared to 2015. This is in line with a Norwegian study that reported that sleep duration among adolescents in Norway from Monday to Friday has declined [22]. The reasons behind our finding may be complex, but one interpretation is related to an increased use of electronic devices in this age group [44]. Norwegian students commonly use digital tablets and this may affect sleep deficits. An increased use of electronic devices (e.g., cell phones, tablets, videogames), especially before bedtime, have been reported to increase the risk of short sleep duration among Norwegian 16–19-year-olds [44]. A study of Taiwanese adolescents investigated the relationship between disturbed sleep due to social media use and academic performance, and found a significant correlation [45].

We further found that sleep deficit among the students was associated with lower school achievements in both mathematics and science. Moreover, it helped explain the decrease in achievement in mathematics and science and has, to our knowledge, not yet been assessed in Norway. Sivertsen et al. suggested that sleep should be a factor to consider when educational difficulties are observed among 16–19-year-olds [46]. They found that short sleep duration increased the risk of poor academic achievements, using grade point average in national tests in mathematics and two other subjects [46]. Their results are somewhat related to our findings, but we assessed students’ sleepiness at school, not their sleep duration. Both short sleep duration and non-optimal sleep patterns have shown increased risk for poor school achievement internationally [47]. They found that, after adjusting for sociodemographic information, short sleep duration and sleep deficit were the sleep measures with the highest odds of poor academic achievements.

The fact that adolescents spend increasingly more time on electronic devices [44] and also report feeling sleepy at school constitutes a major public health concern as well as a concern for their academic achievements. Chronic sleep loss and associated sleepiness and daytime impairments in adolescence are a serious threat to academic achievements and health [47].

Associations between sleep deficit and school achievement were just slightly reduced when adjusting for SES. The same also applies for the explanation of negative changes in the subjects’ scores. Since both sleep and school achievement are socioeconomically patterned [24], we could anticipate that these associations were mostly caused by socioeconomic differences. When this was not the case, our findings can be viewed as being even stronger, suggesting that sleepiness and sleep deprivation should be addressed in all groups of students, not only the socially disadvantaged.

Not only was there a negative development in sleep deprivation over time, students’ academic achievements were also worse than their other Nordic counterparts in 2019 than they were in 2015. In 2019, the Norwegian students’ achievements in science were as much as 1 year behind Swedish students and 2 years behind Finnish students. These differences have, until now, been explained solely from an educational perspective, focusing on fewer hours of instruction in science compared to other Nordic countries [6]. National researchers and policymakers have discussed ways to improve this. However, the current results, point to negative health behavior as an explanation for this negative change. We have recently reported that this negative change in academic outcome is partly explained by the students’ failure to have breakfast [19]. Our current results show that, for both mathematics and science, sleep deficits may also explain some of the negative development. As much as half a year of schooling can be explained by sleep deficits. Over time, such behavior can have detrimental impacts on Norwegian students’ competences and capacities. Our results call for action, not only from the educational and health systems, but also politicians willing to address this issue. Sleep is a factor in the private sphere, with parental responsibility playing a big part, however, our results show that it affects the learning capacity of Norwegian students and should therefore be discussed outside of the private sphere. This includes a discussion of recommendations for sleep duration and how to disseminate such recommendations. An important learning point from our findings in this study is that health behaviors are important for academic achievements and should, therefore, be discussed in the education system.

### Strengths and limitations

The strengths of our study include the use of representative samples, thereby enabling its generalizability to the population. This is important for educational stakeholders and policy. Furthermore, the data is subject to strict quality assurances, including piloting of all data, having a number of researchers around the globe working on the theoretical foundations, reliability, and validity of both the questionnaires and tests. Psychometricians further ensure comparability across cultures and time, as well as reliability and validity of all scales. The achievement scales are calibrated to ensure that they are comparable from one cycle to the next. The TIMSS is a cross-sectional study and therefore causality cannot be inferred. However, there are causal methods that can be used to enhance the level of possible causality [48]. We used this approach to explain time changes in achievement. We may still not draw causal inferences; however, the approach allows for more robust and valid results. Furthermore, we also validated our findings by triangulating across subject domains, time points, and students’ and teachers’ ratings.

The item of sleepiness (reported by students) was not asked in 2015, but was added in 2019. This is a limitation in the study. Also, the teachers reported whether the students sleep deficits limited their teaching in class, and not if the students had sufficient amount of sleep. Hence, this item is an indirect measure, i.e., a limitation. Having a scale with several items measuring sleepiness and sleep deficit, would have been ideal and enhanced the validity. This is not available in the TIMSS data. However, the comparison across time within the same country increases the validity. Another limitation is that the questions regarding sleepiness and sleep deficit are related to the quantity of sleep instead of the quality of sleep. However, using different methods, and having both teacher and student ratings available, the results arrived at the same conclusion. Regarding measurements of sleep, actigraphic measures would be best, but this was not feasible with about 8000 students responding to mathematics and science tests. A randomized, controlled trial would be the preferred design to identify causal relations between sleepiness and school achievement. However, this is not ethical and our results with the strengths mentioned above explain the best available design, in our opinion. Future research should also consider moderation effects. For example, students with lower SES may be at risk for poorer academic achievement, and sleep deficits may exacerbate this risk.

## Conclusions

There has been an increase in sleep deficits among Norwegian 9th grade students from 2015 to 2019. A significant negative association was found between sleep deficit and school achievements in both mathematics and science, both in 2015 and 2019, adjusted for gender, SES, and minority status. Mediation analyses revealed that sleep deficit explained part of the decline in school achievement. Sleep deficits among adolescents may have negative public health implications. The representative sample, the quality-assured data, the triangulation, and robust methodology have provided a unique opportunity to emphasize the importance of sleep for students’ academic achievements.

## Data Availability

The TIMSS data are publicly available on https://timss2019.org/international-database/?_gl=1*17arw7q*_ga*MjEzNTQyNzE3Mi4xNTM0NjY2Mzcx*_ga_L2FMXN42HR*MTY0NzI4NzUyNy4yMC4wLjE2NDcyODc1MjcuMA.

## Abbreviations

TIMSS: Trends in International Mathematics and Science Study
SES: Socioeconomic status
